# Results of endoscopic transcanal tympanoplasty performed by a young surgeon in a secondary hospital^☆^

**DOI:** 10.1016/j.bjorl.2018.12.012

**Published:** 2019-02-28

**Authors:** Mert Cemal Gokgoz, Hamdi Tasli, Bekir Helvacioglu

**Affiliations:** a Siirt State Hospital, Department of Otolaryngology, Siirt, Turkey; b Sanliurfa Birecik State Hospital, Department of Otolaryngology, Sanliurfa, Turkey

**Keywords:** Tympanoplasty, Tympanic membrane, Otitis media, Timpanoplastia, Membrana timpânica, Otite media

## Abstract

**Introduction:**

Tympanoplasty is performed to close the tympanic membrane perforation and recover the hearing level of patients with non-suppurative chronic otitis media. Endoscopic tympanoplasty has recently been increasingly preferred by ear nose and throat surgeons to treat tympanic membrane perforations.

**Objective:**

The aim of this study is to discuss the outcomes of patients undergoing endoscopic tympanoplasty performed by a young surgeon in a secondary hospital in the context of the literature.

**Methods:**

Fifty patients undergoing endoscopic Type 1 tympanoplasty between February 1, 2017 and February 1, 2018, were included. The patients’ age, gender, perforation side and size, preoperative and postoperative pure tone audiometry, graft failure, postoperative pain and complication status were evaluated.

**Results:**

The graft success rate was 94% at 6 months postoperatively. Audiometry thresholds were obtained at frequencies of 0.5, 1, 2 and 4 kHz. Preoperative pure tone audiometric thresholds were 41.6, 36.3, 34.1, and 39.1 dB, and postoperative, 6 months after surgery, 19.5, 17.8, 17.5, and 20.8 dB. Pure tone audiometry air-bone gaps at the same frequencies changed from 30.5, 24.6, 22.2, and 28.6 dB preoperatively, to 11.0, 9.3, 8.6, and 13.9 dB 6 month after the surgery. There was a statistically significant improvement between the preoperative and postoperative pure tone audiometry, and air bone gaps at all measured frequencies (*p* < 0.05).

**Conclusion:**

Endoscopic transcanal cartilage tympanoplasty has become more commonly performed by otolaryngologists due to the shortening of operation and hospitalization times as well as similar audiological results to those obtained with microscopic tympanoplasty. The surgical and audiological results of a young ear nose throat specialist can reach a similar level of success to those of experienced surgeons, due to a fast learning curve.

## Resumo

**Introdução:**

A timpanoplastia é realizada para fechar a perfuração da membrana timpânica e restaurar a audição de pacientes com otite média crônica não-supurativa. Recentemente, a timpanoplastia endoscópica tem se tornado a técnica preferida por cirurgiões otorrinolaringologistas, com indicação crescente em casos de perfurações timpânicas.

**Objetivo:**

O objetivo deste estudo é discutir os resultados em pacientes submetidos a timpanoplastia endoscópica realizada por um jovem cirurgião em um hospital secundário, no contexto da literatura.

**Método:**

Cinquenta pacientes submetidos a timpanoplastia endoscópica Tipo 1 entre 1° de fevereiro de 2017 e 1° de fevereiro de 2018 foram incluídos. A idade dos pacientes, sexo, lado e tamanho da perfuração, limiares da audiometria tonal pré-operatória e pós-operatória, falha do enxerto, dor pós-operatória e ocorrência de complicações foram avaliados.

**Resultados:**

A taxa de sucesso do enxerto foi de 94% aos 6 meses de pós-operatório. Nas frequências de 0,5, 1, 2 e 4 kHz, a audiometria tonal pré-operatória mostrava limiares de 41,6; 36,3; 34,1 e 39,1 dB e a pós-operatória após 6 meses, revelou limiares de 19,5; 17,8; 17,5 e 20,8 dB. Nas mesmas frequências, os gaps aéreo-ósseos pré-operatório na audiometria tonal eram de 30,5; 24,6; 22,2 e 28,6 dB e com 6 meses de pós-operatório, de 11,0; 9,3; 8,6 e 13,9 dB. Houve melhora estatisticamente significante entre os limiares da audiometria tonal pré- e pós-operatória em todas as frequências (*p* < 0,05). Houve diferença estatisticamente significante entre os gaps aéreo-ósseo pré- e pós-operatório, ocorrendo diminuição dos mesmos em todas as frequências (*p* < 0,05).

**Conclusão:**

A timpanoplastia endoscópica com cartilagem por via transcanal tem sido mais comumente realizada pelo otorrinolaringologista devido ao menor tempo de cirurgia e hospitalização e resultados audiológicos semelhantes aos com o uso de microscópico. Os resultados cirúrgicos e audiológicos de um jovem especialista em otorrinolaringologia podem atingir um nível semelhante ao de cirurgiões experientes, com uma rápida curva de aprendizado.

## Introduction

Tympanoplasty is performed to close tympanic membrane perforations and restore the hearing level in non-suppurative chronic otitis media. Endoscopic tympanoplasty has recently begun to be preferred by ear nose and throat (ENT) surgeons, with increasing frequency, in cases of tympanic membrane perforations.^1^, ^2^ When comparing the classical approach with a microscope, endoscopic surgery shows some advantages such as less operation time, visualization of the whole surgical field with no need of changing the angle or the patient position as usually occurs when a microscope is used, ability to operate with a minimal incision, short postoperative hospitalization time, and low pain levels.^3^, ^4^ Angled endoscopes are an important advantage for the surgeon to visualize some areas that are commonly difficult to see with a microscope, such as the anterior quadrant of the tympanic membrane, sinus tympani and facial recess.^5^ The aim of this study is to discuss the outcomes for patients undergoing endoscopic Type 1 tympanoplasty performed by a young surgeon in a secondary hospital in the context of the literature and evaluate both the performance of the young surgeon and the effectiveness of the endoscopic method.

## Methods

The study was approved by Kecioren Training and Research Hospital Ethics Committee (approval no. 2018/1730), and informed consent was obtained from all participants included in the study. Fifty patients undergoing endoscopic Type 1 tympanoplasty between February 1, 2017 and February 1, 2018, were included. All operations were performed by the same surgeon. The patients’ age, gender, perforation side and size, preoperative and postoperative pure tone audiometry, graft failure, postoperative pain and complication status were evaluated. Postoperative audiometry tests were performed 6 months after the operation, and the pure tone audiometry were obtained at 0.5, 1, 2, and 4 kHz.

The criteria for admission to the study were the absence of ear drainage for the last 3 months prior to surgery, no evidence of inflammation, and absence of ossicular pathology and mastoiditis on the computed tomography (CT) images of the temporal bone. Endoscopic fat myringoplasty, butterfly myringoplasty and ossicular repair operations were considered as exclusion criteria.

All patients were operated under general and hypotensive anesthesia using 0° and 30° endoscopes of 4 and 2.7 mm in diameter (Karl Storz, Tuttlingen, Germany). The combination of adrenaline and lidocaine (Jetokain Adeka, Samsun, Turkey) was used to infiltrate the tragal cartilage graft site and the four quadrants of the external ear canal. The operation time was recorded and the registration started when the local anesthesia was initiated. The surgical step of filling the external ear canal with spongostan was included in the duration of the operation. The perforation edges on the tympanic membrane were refreshed by scraping its margins. The tympanomeatal flap was elevated with an incision made from 4 to 6 mm lateral to the annulus. Adrenaline- impregnated cotton was used to reduce the bleeding and facilitate the dissection during the elevation. After the flap elevation, the tympanic membrane was removed from the malleus. Subsequently, a 5 mm incision was made from the posterior part of the tragus for tragal cartilage retrieval. The tragus apex was protected to prevent cosmetic deformation. The graft area was sutured with 4.0 rapid-absorbing Vicryl. Perichondrium was left on one side of the cartilage graft, and a pocket was opened to accommodate the manubrium mallei on the tragal cartilage. The cartilage piece was placed under the elevated tympanic membrane, over the malleus and the pocket on the graft accommodated the manubrium mallei (over-underlay technique). The tympanomeatal flap was laid back into the canal and whether the perforation was completely closed was then checked. The external ear canal was then filled with spongostan. The patients were discharged on the first postoperative day.

**Figure 1:**
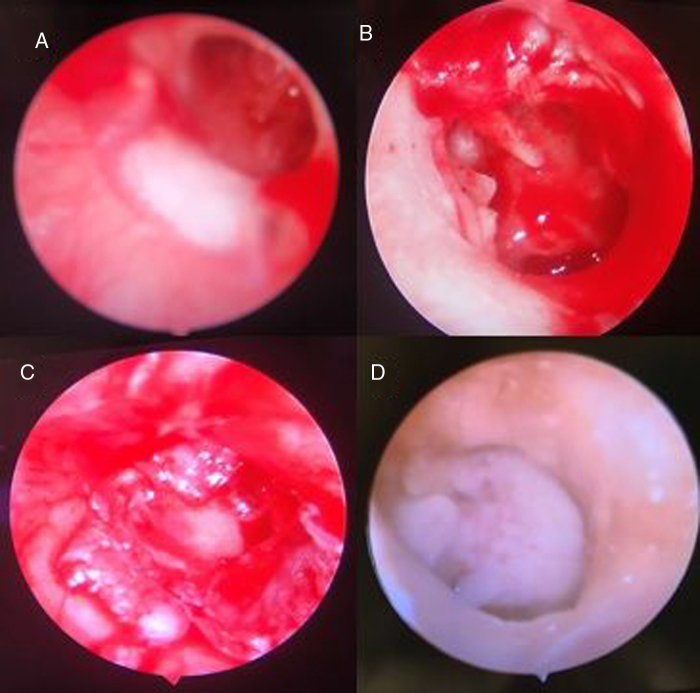
(A) Pre-operative endoscopic view of the tympanic membrane, (B) the surgical field after elevation of the tympanomeatal flap, (C) endoscopic view of the tympanic membrane after grafting, (D) a post-operative endoscopic view of the graft at three months.

Fig. 1 shows (A) pre-operative endoscopic view of the tympanic membrane, (B) the surgical field after elevation of the tympanomeatal flap, (C) endoscopic view of the tympanic membrane after grafting, (D) a post-operative endoscopic view of the graft at three months.

All statistical analyses were performed with SPSS 20.0 (IBM Co., Armonk, NY, USA). Patients’ characteristics, such as age and gender, perforation side and size, preoperative and postoperative pure tone audiometry, graft success rates, and duration of surgery were analyzed using the Student's and paired-sample *t*-tests. Statistical significance was accepted as *p* < 0.05.

**Figure 2:**
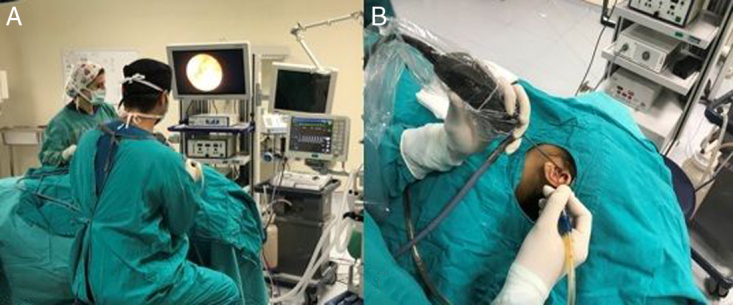
(A) The operation room placement: the monitor is at the eye level of the surgeon and the nurse at the opposite side, (B) the endoscope is on the left hand and the surgical instrument on the right.

Fig. 2 shows (A) the operation room placement: the monitor is at the eye level of the surgeon and the nurse at the opposite side, (B) the endoscope is on the left hand and the surgical instrument on the right.

## Results

Fifty patients were included in the study. Sixteen (32%) were female and 34 (68%) were male. The average age was 22.12 years, and the age range was 14–40 years. Thirty-two (64%) patients had surgery on the left ear, and 18 (36%) had it on the right. The preoperative perforation dimension was 5.3 mm on average on the longitudinal axis. The graft success rate was 94% on endoscopic and otoscopic examination at 6 months postoperatively. As a postoperative complication, three patients had a crescent-shaped perforation in the anterior quadrant. In patients with graft failure, the preoperative perforation size was measured as 5.7 mm in the longitudinal axis, and no relationship was found between the perforation size and graft failure. Two patients presented swelling and pain in the graft area, both improved with medical treatment (Table 1).

**Table 1:** Number of patients, age, perforation size, graft closure rate and complications by sex.

	Male	Female	Total
Number of patients	34 (68%)	16 (32%)	50 (100%)
Age (years) (average–range)	22.23 (15–40)	21.87 (14–36)	22.12 (14–40)
Perforation size (mm) (longitudinal axis)	5.29	5.33	5.3
Graft closure rate	94.11%	93.75%	94%
Complications			
Residual perforation	2 (5.89%)	1 (6.25%)	3 (6%)
Swelling and pain	2 (5.89%)	–	2 (4%)

Sixteen perforations were medium-sized (25%–50% of all tympanic membrane) and 34 were large (greater than 50%). The mean duration of operation was 65 ± 10 min (range, 52–80 min).

**Figure 3:**
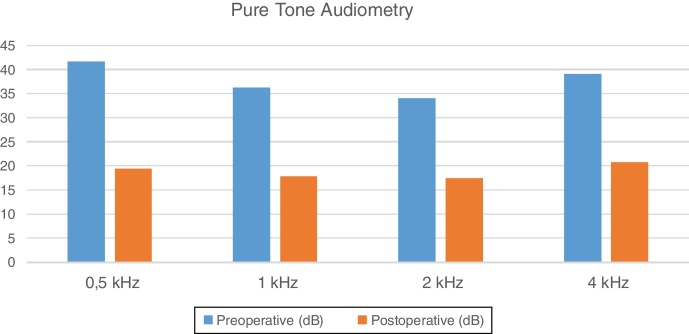
Results of pure tone audiometry.

Preoperative and 6 months postoperative pure tone audiometry at frequencies of 0.5, 1, 2 and 4 kHz, showed thresholds of 41.6, 36.3, 34.1, and 39.1 dB; and 19.5, 17.8, 17.5, and 20.8 dB respectively (Fig. 3).

**Figure 4:**
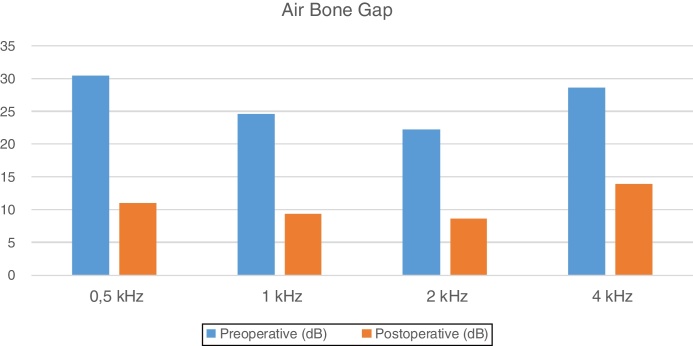
Results of air bone gap.

Preoperative and 6 months postoperative air bone gaps at the same frequencies, were 30.5, 24.6, 22.2, and 28.6 dB and 11.0, 9.3, 8.6, and 13.9 dB respectively (Fig. 4).

There was a statistically significant improvement between the preoperative and postoperative pure tone audiometry values at all frequencies (*p* < 0.05). There was a statistically significant reduction between the preoperative and postoperative air bone gap values at all frequencies (*p* < 0.05).

## Discussion

The use of endoscopes in otologic surgery has many advantages. The microscope is still considered the gold standard for ear surgery, but the use of endoscopic methods is increasing, especially for diagnostic procedures and basic tympanoplasties. Endoscopes, which in recent years were only employed for diagnostic purposes,^1^ have come into use in surgical procedures, and the surgical procedures have become less invasive.^2^, ^3^, ^4^, ^5^ The duration of the surgery has been shortened and patient's comfort has been improved by decreasing the postoperative pain and the need for dressing.^6^ Manipulation of the microscope and positioning of the patient's head in microscopic surgeries has become unnecessary, since the manipulation of the endoscope can be easily achieved by the surgeon. Another advantage of the endoscope is that the entire surgical field can be seen on one screen. Angled endoscopes can be an important advantage for the surgeon to visualize some areas that are considered difficult-to-see areas when using a microscope, such as the anterior quadrant of the tympanic membrane, sinus tympani, and facial recess.^5^ In microscopic tympanoplasty, usually the approaches are via postauricular or endaural incisions, but in endoscopic transcanal surgeries, the tympanomeatal flap elevation is performed as a minimally invasive procedure, producing better cosmetic results.

In terms of graft success, endoscopic and microscopic methods are employed with similar rates.^7^ Both methods result in similar results when it comes to improving the pure audiometry and reducing the air bone gap. In our study, perforation dimensions were measured as 5.3 mm on the longitudinal axis on average, and the graft success rate was 94%. Three patients had a crescent-shaped perforation in the anterior part of the graft. The audiological results were only partially improved in patients who had graft failure. Postoperative graft failure may have happened due to an inadequate support of the spongostan®, inadequate patient blood supply, or infection.

The duration of the operation was registered in our study, and in the final 10 cases, the surgery lasted less than 60 min. Tseng et al.^8^ reported that the operation time stabilized below 60 min after the 150^th^ patient in their study. Dogan et al.^9^ measured the mean duration of operation as 88.60 ± 21.10 min in the first 30 patients, 62.00 ± 12.48 min in the second 30 patients, and 43.81 ± 8.34 min after 60 patients. In our study group, the duration of operation was over 70 min in the first 10 patients, and it stabilized below 60 min in the last 10 patients. The most time-consuming surgical step was assessing the elevation of the tympanomeatal flap and the annulus, and also the graft preparation stage. The training of the surgeon to perform endoscopic operations was based on the microscopic tympanoplasty learning. The 50 patients included in the study represent the first 50 patients that the surgeon operated after completing his assistant education. All 50 patients’ surgeries were performed using the endoscopic technique. No microscopic intervention was needed for any surgical step, and all the operations were completed via transcanal with the endoscopic method.

When frequency-based differences were evaluated, statistically significant improvements were observed at frequencies of 0.5, 1, 2, and 4 kHz in both, the pure tone audiometry and air bone gap values. At 1 and 2 kHz frequencies, the air bone gap was reduced to less than 10 dB. At frequencies of 0.5, 1, and 2 kHz, the pure tone audiometry values were reduced to less than 20 dB postoperatively. Although a statistically significant improvement was observed at 4 kHz, the reason for the lesser improvement compared with other frequencies was attributed to the cartilage-grafting method. In addition, previous studies have reported that there is no difference in terms of audiological results between fascia and cartilage,^10^, ^11^ another study reported that lesser gain was detected at 4 kHz than at the other frequencies.^12^ Compliance features such as stiffness or thickness of cartilage graft and weakness in the acoustic transmission, may lead to this result. An increase in the size of the cartilage graft with an increasing perforation size may have created a load on the manubrium mallei. In three cases where the perforation was not completely closed, partial audiological improvement was achieved, and no hearing aid was needed in any case.

Endoscopic surgery also has some disadvantages, such as the necessity of holding the endoscope continuously in one hand; working with only one instrument; the need for hypotensive anesthesia for decreasing hemorrhage, especially in the tympanomeatal flap elevation part; two-dimensional imaging; and decreased feeling of depth. Working with one hand, requires a bloodless surgical site. If the surgical site is bleeding, the surgeon will have to deal with both aspiration and flap elevation at the same time. Because of this, especially the necessity of hypotensive anesthesia and use of adrenaline soaked cotton during flap elevation are important. In the microscopic method, the surgeon can perform both aspiration and surgical procedures simultaneously. The above-mentioned endoscopic surgical technique should be preferred, especially in cases of simple tympanoplasty without ossicular involvement. In all cases in the study, the external ear canal has sufficient width and flatness. Endoscopic methods are less easily performed in cases accompanied by diseases narrowing the external ear canal, such as craniofacial anomalies and Down syndrome/Goldenhar syndrome etc.^13^ At the same time, the risk of traumatic mechanical damage due to the use of the endoscope during operation and the thermal effect of the xenon light source can be mentioned. Kaya et al.^14^ stated that cochlear functions stabilized after endoscopic transcanal tympanoplasty with a cold light source. Kozin et al.^15^ recommended decreasing the light intensity, changing the position of the endoscope frequently, and removing the endoscope to allow tissue cooling. In the endoscopic method, the short duration of the procedure reduces the risk of light damage for both the surgeon and the patient. The main risky process for light damage was assessed as after the tympanomeatal flap and annulus elevation. Promontorium is under direct light especially in removing the tympanic membrane from the malleus and this takes about 5 min. The endoscope is constantly moving in the hand of the surgeon and the removal of the endoscope from the canal for cleaning are the other reasons that may reduce this risk.

## Conclusion

In conclusion, endoscopic transcanal cartilage tympanoplasty has become more commonly performed by ENT specialists due to the short operation and hospitalization time and similar audiological results to those of the microscopic method. The surgical and audiological results of a new ENT specialist can reach a similar level to those of experienced surgeons, with a fast learning curve. Increased patient comfort has also led patients to ask for an endoscopic approach. It should be noted that the microscopic method is the surgical gold standard and that the above-mentioned endoscopic method is particularly suitable in cases of Type 1 tympanoplasty, which involves a wide and flat external ear canal.

## Conflicts of interest

The authors declare no conflicts of interest.
